# A predictive model for daily cumulative COVID-19 cases in Ghana

**DOI:** 10.12688/f1000research.52403.2

**Published:** 2022-03-04

**Authors:** Abdul-Karim Iddrisu, Emmanuel A. Amikiya, Dominic Otoo

**Affiliations:** 1 Mathematics and Statistics, University of Energy and Natural Resources, Sunyani, Ghana; 2 Department of Management Science, Ghana Institute of Management and Public Administration, Accra, Ghana

**Keywords:** Covid-19, forecasts, generalized additive models, polynomials and spline models.

## Abstract

**Background: **Coronavirus disease 2019 (COVID-19) is a pandemic that has affected the daily life, governments and economies of many countries all over the globe. Ghana is currently experiencing a surge in the number of cases with a corresponding increase in the cumulative confirmed cases and deaths. The surge in cases and deaths clearly shows that the preventive and management measures are ineffective and that policy makers lack a complete understanding of the dynamics of the disease. Most of the deaths in Ghana are due to lack of adequate health equipment and facilities for managing the disease. Knowledge of the number of cases in advance would aid policy makers in allocating sufficient resources for the effective management of the cases.

**Methods:** A predictive tool is necessary for the effective management and prevention of cases. This study presents a predictive tool that has the ability to accurately forecast the number of cumulative cases. The study applied polynomial and spline models on the COVID-19 data for Ghana, to develop a generalized additive model (GAM) that accurately captures the growth pattern of the cumulative cases.

**Results: **The spline model and the GAM provide accurate forecast values.

**Conclusion: **Cumulative cases of COVID-19 in Ghana are expected to continue to increase if appropriate preventive measures are not enforced. Vaccination against the virus is ongoing in Ghana, thus, future research would consider evaluating the impact of the vaccine.

## 1. Introduction

Three months after the emergence of the coronavirus (SARS-CoV-2) in China, about 118,000 confirmed cases and 4,291 associated deaths were reported globally. The disease spread so rapidly that in less than half a year, the World Health Organization (WHO) declared it a global pandemic. ^19, 32, 33^ As of February 24, 2021, about 112,741,607 cases have been reported globally with 2,498,533 associated deaths and 88,310,527 recoveries. Africa is the least affected continent with about 3,872,085 cases, 102,286 deaths and 3,421,548 recoveries. ^11^ Currently in Ghana, a total of 80,759 cases have been reported with 582 deaths and 73,365 recoveries. ^31^

However, various governments, health stakeholders and policy makers have introduced measures to either prevent the spread or manage the confirmed cases. Some of the preventive measures include “lockdown”, frequent washing of hands under running water with soap, to avoid touching the face, wearing of nose masks at public places, disinfection of hands and surfaces with alcohol-based sanitizer, and observing physical social distance. ^33^ Some of the management measures include the provision of treatment facilities, equipment, recruitment of health professionals and provision of incentives to frontline workers.

Despite the preventive and management measures proposed and implemented by various governments and stakeholders, the disease is still spreading at an alarming rate. For instance, by April 7, 2020, Africa only registered about 10,268 confirmed cases, with 491 deaths. ^11^ Compared to the current statistics for Africa, it is clear that the spread is surging. This surge can be observed in Ghana and many other countries in the world. The surge in registered cases and associated deaths implies that the preventive and management measures are not effective. This further implies that the current understanding of the complete dynamics of the disease is lacking. Most of the death cases in Ghana are due to lack of adequate health personnel, equipment and facilities for managing the disease. Knowledge of the predicted future number of cases in advance would aid policy makers in allocating sufficient resources for the effective management of the cases. Hence, a predictive tool is necessary for the effective management and prevention of the cases. Therefore, the development of accurate statistical and mathematical models are necessary for the effective management and prevention of coronavirus disease 2019 (COVID-19), as the models are able to forecast future events.

Effective policies against the virus can be developed from the inferences of data, modeling, and scientific findings including vaccines. ^15^ Indeed, a lot of effort has been made by scientists, epidemiologists and even economists in their research in order to better understand the dynamics of COVID-19. Some COVID-19 vaccines are ready for use and other vaccines are at different phases of clinical trials. Apart from the development of vaccines, many governments are working tirelessly to ensure the availability of resources such as funds and data repositories to assist researchers. ^19^ In Africa, the screening and vaccination of patients with an experimental vaccine developed by Novavax started on August 17, 2020 in South Africa. ^21^ This trial received an amount of USD 15 million in funding from the Bill and Melinda Gates Foundation. ^21^ More information on the pandemic can be found at. ^12, 21, 22^

Furthermore, some researchers ^33^ have investigated how information from social and behavioral science can be used to ensure that human behavior are in line with the COVID-19 safety protocols outlined by epidemiologist and public health experts. Tsallis and Tirnakli ^30^ studied and predicted the peak of COVID-19 cases around the world by proposing a q −statistical functional form which provides a satisfactory description of the available data for all countries. ^30^ Higher COVID-19 morbidity and mortality is associated with elderly people. ^8^ Milani ^24^ researched the interconnectedness of countries and how this influences the spread of the virus. The authors estimated the vector autoregression (VAR) model using data on existing social networks across countries, and showed that social networks can be used to explain the spread of the virus as well as the spread of perception in risk and social distancing behavior across countries. Some researchers ^26^ have developed simple COVID-19 epidemic models to explore strategies on how to control the pandemic. The authors ^2^ have assessed and compared the pattern of the virus in Nigeria and seven other countries using data on the first 120 days of the pandemic. Similar patterns of COVID-19 spread have been observed in Egypt, Ghana, and Cameroon. ^2^

The emergence of the COVID-19 virus has led to the development and applications of various mathematical and statistical modeling approaches to study the dynamics, predict and forecast. A systematic review aimed at summarizing trends in the modeling approaches used for predicting and forecasting has been carried out in. ^14^ The main aim of their discussion was to examine the accuracy and precision of predictions. They achieved their goal by “ *comparing predicted and observed values for cumulative cases and deaths as well as uncertainties of these predictions*”. ^14^ The most commonly used models in the study and predictions are the compartmental model, susceptible-infected-recovered (SIR) and susceptible-exposed-infectious-recovered (SEIR), statistical models, growth models and time series, artificial intelligence models, Bayesian approaches, network models, and agent-based models. ^14^ The studies revealed that Bayesian models are more accurate relative to the classical statistical models. Bayesian methods have the ability to give better predictions even with small data sets. The study showed a significant negative correlation between the predictions, the observed values and the time period used in the modeling. This indicates that, with longer time periods used, models are likely to produce more accurate estimates.

Predictive models ^20^ employed to study spatial-temporal patterns of the pandemic in Africa showed variability in time and space across the study domain. A cubic model that is more robust in predicting the confirmed cases and deaths was found to be the best performing model relative to other exponential models. ^20^ The study placed much emphasis on the need to encourage self-isolation in other to prevent the spread of the virus. ^20^ Some other modeling approaches include fractional-order derivative-based modeling, ^1^ stochastic meta-population models to estimate the global spread of the virus, ^3^ and a mathematical model that assessed the imposition of the lockdown in Nigeria. ^6^ Various authors have applied the decomposition and ensemble model to forecast COVID-19 confirmed cases, deaths, and recoveries in Pakistan. ^36^

Researchers have studied the dynamics of COVID-19 in Ghana, although more research still needs to be conducted. Geospatial technologies ^28^ have been applied to the COVID-19 data in Ghana, to study the trend of the cases and model the near future trends in Ghana. This study found higher cases of the virus in areas with higher population densities which are in the southern part of the country. ^28^ The authors in ^5^ studied the “human-environment-human” using “mathematical analysis and optimal control theory”. Their results showed that adhering to safety measures “such as practicing proper coughing etiquette, covering the nose/mouth with tissues/cloth when coughing or sneezing, and washing of hands after coughing or sneezing by both asymptomatic and symptomatic subjects are the most cost-effective measures”.

Other researchers studied the relationship between urban planning and public health to support decisions and policies in the “fight” against the virus. ^4^ They also looked at how we can leverage on the pandemic to build healthier cities since currently, only a few Ghanaians live in well-planned settlements and majority of Ghanaians are susceptible to the pandemic due to their less hygenic environments. ^4^ Growth curves and generalized additive models (GAMs) have been used to assess whether the basic reproductive number of COVID-19 is different across countries and to determine factors that increase the level of an individual’s vulnerability to the virus. ^18^ Various authors have modeled, predicted and forecast cumulative cases of COVID-19 to study the dynamics of cumulative cases over a period of time. ^37– 42^ The authors in ^42^ used cumulative covid-19 data and time series models to forecast the epidemiological trends of COVID-19 pandemic for top-16 countries where 70%–80% of global cumulative cases are high. Also, a deep learning ensemble approach has been adapted by the authors in ^41^ to determine the best auto-regressive integrated moving average (ARIMA) model for predicting and forecasting cumulative COVID-19 cases across multi-region countries. Nonlinear growth models such as the Gompertz, Richards, and Weibull were implemented to cumulative covid-19 data in order to study the daily cumulative number of COVID-19 cases in Iraq. ^40^ Bartolomeo et al. ^43^ applied the exponential decay model (EDM) to estimate and forecast the cumulative number of COVID-19 infections in Italy. These authors compared the EDM and the Gompertz model. The exponential decay model applied to the weighted and averaged growth rates appears to be better than growth models such as Gompertz’s for modeling the number of cases of the COVID-19. In this study, linear, polynomial and generalized linear models (GLMs) are employed to explain the growth pattern of the number of cumulative cases of COVID-19 and also, to predict and forecast the number of cumulative cases in Ghana. These models were implemented to the Ghana COVID-19 data and compared for best model selection and results discussed and conclusion drawn.

## 2. Methods

**Figure 1.**
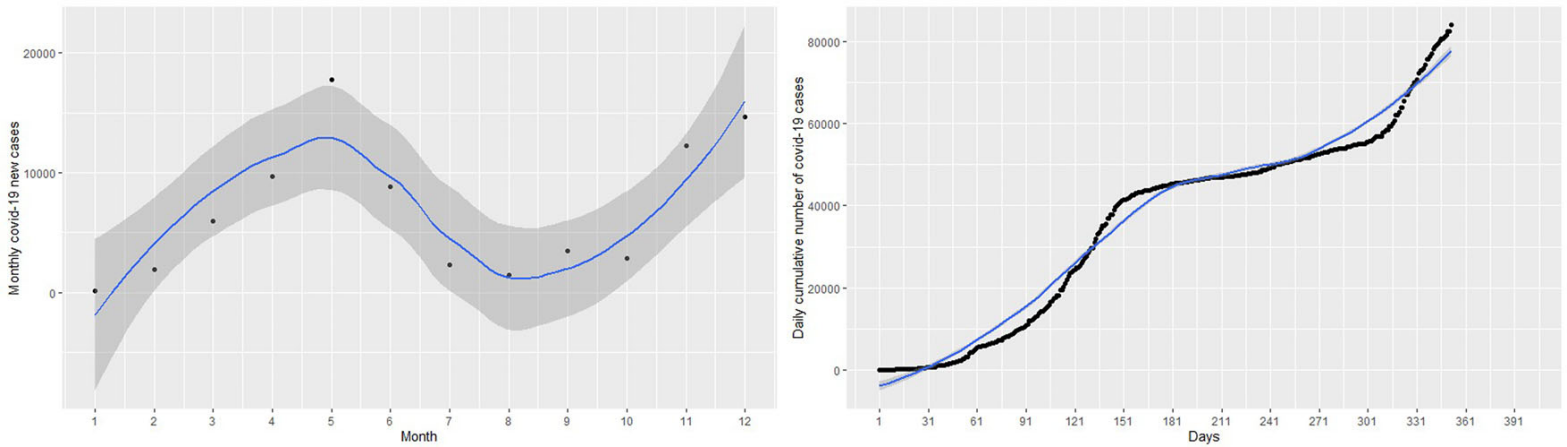
Plots of monthly new coronavirus disease 2019 (COVID-19) cases (left panel) and daily cumulative cases (right panel) from March 14, 2020 to February 28, 2021.

This section discusses statistical methods that have the ability to capture and explain the non-linearity in the number of cumulative COVID-19 cases shown in Figure 1B. There are situations where the relationship between the response variable and the predictor are non-linear. Thus, the linear regression models do not yield accurate statistical inferences due to their inability to capture non-linearities. There are methods that can be used to modify the linear regression model to enable them capture non-linear effects. Such modifications lead to polynomial regression, spline regression, and GAMs that are accurate for modeling non-linear relationships between responses and predictor. ^9, 16, 34^

The polynomial regression approach extends the linear regression to capture non-linearity by including terms of higher order such as squares or cubes in the linear regression model. Spline regression on the other hand fits a smooth curve characterized by a series of polynomial segments. The spline segments are delimited using values called knots. The GAMs are used to fit spline models with an automatic selection of knots.

In the following sections, the polynomial, spline and GAM methods are discussed in detail. The aim is to apply them to model the COVID-19 cumulative cases, so that, the most accurate models will be used to forecast future events. Root mean square errors ( *RMSE*), R−square (R ^2^), and Akaike information criterion (AIC), mean absolute error (MAE), and mean absolute percentage error (MAPE) ^44^ will be used to assess the accuracy of the models. The *RMSE* is the model prediction error which is the average difference in the observed and predicted outcome values. The R ^2^ on the other hand represents the square of correlation between the observed and predicted outcome values or the amount of explained variability in the data. The MAE is the average of all absolute errors and MAPE is the absolute percentage of errors forecasts and is used to measure of accuracy of the forecasts. ^44^ The most accurate model is the model with the lowest RMSE and AIC, MAE, MAPE, and the highest R ^2^. When a study involves small sample size, the PIC criterion ^36^ can be used. The PIC criterion takes into account a larger penalty from adding too many regression parameters and when the sample size is small. ^36^

### 2.1. Polynomial regression model

Given the plot of the cumulative cases of the COVID-19 in Figure 1A, it is obvious that linear regression models

$$y_{i}=\beta_{0}+\beta_{1}x_{1}+\epsilon_{i}$$

do not provide accurate statistical inferences since the relationship between the observed cumulative cases and time (in days) is non-linear. There is the need to modify the linear model to account for the non-linear relationship, by using polynomials of higher degree (i.e. degrees greater than one). In general, non-linear effects can be modelled by using polynomials of degree *p* defined as follows: ^27^

$$\begin{array}{l} y_{i}=\beta_{0}+\beta_{1}s_{i}+\beta_{2}s_{i}^{2}+\beta_{3}s_{i}^{3}+\ldots +\beta_{p}s_{i}^{p}+\epsilon_{i}, \end{array}$$ (1)

where *y _i_* is the response variable, *β _j_* for *j* = 0,…, *p* are parameters, *s _i_* is a basis function of the predictor *x _i_*, defined for all *i* as follows:

$$s=\left(\begin{array}{lllll} 1 & x_{1} & x_{1}^{2} & \ldots & x_{1}^{p}\\ 1 & x_{2} & x_{2}^{2} & \ldots & x_{2}^{p}\\ \vdots & \vdots & \vdots & & \vdots \\ 1 & x_{n} & x_{n}^{2} & \ldots & x_{n}^{p}. \end{array}\right)$$

In the regression model ( 1), the parameters *β _j_* are independent of the predictor variable *x _i_*, however, the basis functions depend non-linearly on the predictor variable. Consequently, the parameters can be estimated using ordinary least squares approaches. In general, we can express model ( 1) in terms of a smooth function as follows:

$$y_{i}=f(x_{i})+\epsilon_{i},$$

where *f* represents a function or a transformation of the predictor variable *x _i_*. ^27^

Polynomial models are easy to implement, however, their non-local property (i.e. the fitted function at any given value *x*_0_ depends on data values that are far from *x*_0_) is their major disadvantage. This issue can be avoided by dividing the domain of *x* into smaller intervals, fitting accurate polynomials in each interval and then finally combining the piecewise polynomial into a global one. ^27^ The domain of *x* is divided into smaller intervals using an arbitrary number/position of points τ known as knots. ^27^ A piecewise continuous model is fitted by specifying the following functions:

$$f_{1}=1,f_{2}=x,f_{3}=(x-\tau_{1})+,f_{4}=(x-\tau_{2})+,\ldots ,$$

with + as a function defined by:

$$\begin{array}{l} u_{+}=\left\{\begin{array}{l} u,\;if\;u>0\;\\ 0,\;if\;u\leq 0\; \end{array}\right. \end{array}$$ (2)

The combination of these sets of functions give rise to a composite function defined as *f*( *x*).

### 2.2. Spline regression

Polynomial regression does not capture the complete non-linear relationship. An alternative, and often superior approach for modeling non-linear relationships is the use of splines. ^7^ A spline can be perceived as a flexible thin strip of wood or metal that can be used to draw smooth curves. ^27^ They require several weights to be placed at certain positions so that the strip of wood would bend according to the number/position of the weights. ^27^ Statistically, splines are used to reproduce flexible smooth curves. ^27^ That is, splines enable smooth interpolation between fixed points, called knots. They are series of polynomial segments strung together. ^7^

Assume that the curve *f*( *X*) evaluates to a single value *y* for each set of predictors *x*, where *x* can be univariate or multivariate. If the set of knots is defined by *τ*_1_ < *τ*_2 _< … < *τ*_u_ in the domain of *X*, *X* ∈ R, then *f*( *X*) is a special polynomial of degree *p*, called a spline.

In modeling studies a smoothness criterion, which states that all derivatives of order less than *p* are continuous, is usually imposed. ^27^ A physical spline is linear beyond the last knot, thus, more constraints are imposed on derivatives of order 2 or greater at the leftmost and rightmost knots. ^27^ Splines which have these extra constraints are known as restricted or natural splines. Flexibility of the curves can be achieved by increasing the number of knots or the degree of the polynomial. However, it is worth noting that increasing the number of knots may lead to over-fitting due to associated high variances. Furthermore, decreasing the number of knots may lead to a rigid and restrictive function that has more bias. ^27^

Let *f* denote any spline function with a fixed knot sequence and a fixed degree p. Since the spline functions are objects in a vector space V, then *f* can be expressed as follows:

$$f(X)=\overset{K+p+1}{\underset{k=1}{\sum }}\beta_{k}B_{k}(X),$$ (3)

where the *B _k_* are a set of basis functions spanning V and *β _k_* are the associated spline coefficients. ^27^ For any *k* knots, there are *k* + 1 polynomials of degree *p* and *p* × k constraints. This leads to ( *d* + 1)( *k* + 1) − *p* × *k* = *d* + *k* − 1 free parameters. ^13, 34^ For natural or restricted splines, there are *k* free parameters. Since *βB* = ( *βA*)( *A* ^−1^ *B*) = δB* and for any non-singular matrix, there are an infinite number of possible basis sets for the spline.

The advantage of the equation ( 3) is that the estimation of *f* reduces to the estimation of the regression coefficients *β _k_.* Specifically, the specification of Model ( 3) indicates that *f* is non-linear in the predictor but linear in the vector of regression coefficient ***β*** = ( *β*_1_, *β*_2_,…, *β*_*K*+ *p*+1_). One can view the estimation of *f* as an optimization problem that is linear in the transformed variables *B*_1_( *X*),…, *B*_*K*+ *p*+1_( *X*). Consequently, a framework is established for the estimation approaches to be adapted for splines in a wide range of generalized or multivariate regression. ^27^ A more appealing property of spline models is their ability to reduce the estimates to a few regression coefficients. ^27^

Although the flexibility property of splines makes them a better choice for fitting datasets, there are challenges associated with the number of tuning parameters. ^13, 27, 34^ That is, the choice of the basis functions *B* and the degree of the polynomial eventually have little impact. Sauerbrei and colleagues ^27^ noted that spline models are robust to the degree *p* of the polynomial. Polynomials with degree p = 3 (cubic polynomial) are standard because they are smooth curves. If the derivatives of the fitted curves are required, then a higher order polynomial is appropriate. However, the authors in ^27^ have observed that polynomial models with degree *p* > 3 are “effectively indistinguishable”.

Furthermore, modeling with splines involves deciding the number/spacing of knots and whether to use or not use a penalty function (the integrated second derivative of the spline). The absence of a penalty term in the spline model implies the generation of transformed variables which are added to the standard model. Such a procedure where the flexibility of the resulting non-linear function is entirely based on the number of knots is referred to as regression splines. ^27^ If the penalty term is added to the spline modeling, modification of the procedure is required to take into account the penalty term. In that case, each regression function has to be modified separately to obtain smooth splines that exhibit several desirable properties.

Moreover, a discussion on choices of basis *B _k_* functions for splines can be found in. ^27, 34, Chp. 5^ The discussion here will involve *B*-splines and bases that are based on a special parametrisation of a cubic spline. These set of bases depend on the sequence of knots. ^9, 27^ An advantage of the *B*-basis is that the bases have a local support. That is, the *B*-bases are larger than zero in intervals spanned by *p* + 1 knots and zero elsewhere. ^9^ This property of the *B*-bases makes them numerically stable as well as present an efficient algorithm for building the basis functions. ^34^ Detailed information on different types of basis for splines and guidelines for the use of splines can be found in. ^27^

Futher, the selection and placement of knots is challenging due to the arbitrary nature of the task. That is, whenever a non-linear relationship is detected in data, the polynomial terms are not flexible enough to capture the relationship, however, splines require specification of the knots. GAMs provide a tool to automatically fit a spline regression. ^16, 17, 34, 35^ GAMs will be discussed in the section that follows immediately.

### 2.3. GAM

The purpose of this section is to discuss GLMs and their extension to GAMs. The linear models (LMs) are used to model response variables that follow normal distributions whereas GLMs are used to model either normal or non-normal responses. ^25^ The general form of GLMs is:

$$\begin{array}{l} g\left(\mu_{i}\right)=X_{i}\beta , \end{array}$$ (4)

where *μ _i_* = *E*[ *Y _i_*], *g* is a smooth monotonic “link function”, ***X*** is *n* × *p* design matrix of covariates, *X _i_* is the covariate associated with the *i ^th^* subject or item, ***β*** is a 1 × *p* vector of unknown parameters describing the effects of the covariates on the 1 × *n* matrix of responses *Y _i_*, and *n* is the number of observations. The GLMs assumes that the responses *Y _i_* are independent and follow some exponential family of distributions. The exponential family of distributions include Poisson, binomial, gamma, and normal distributions. ^25^ For a detailed discussion of GLMs, see. ^10, 23^ Under the generalized linear mixed (GLMM) effects model, random effect components *Z _i_b _i_* are added to the fixed effect components *X _i_β*, where *b _i_* is 1 × *q* is a vector of random effects and *Z _i_* is a *p* × *q* design matrix of the random effects and $b_{i}∼N\left(0,\sigma^{2}\right),\sigma^{2}$ is the variance of the random effect. So the general form of the GLMM is defined as:

$$\begin{array}{l} g\left(\mu_{i}\right)=X_{i}\beta +Z_{i}b_{i}. \end{array}$$ (5)

GLMs are specified in terms of the linear predictor *η* = *X _i_* ***β*** which is the same as in the linear models. Hence, most of the concepts of linear modeling are maintained under the GLM framework with little modification. The formulation of the model is the same except that one has to choose the link function and the distributional assumption of the data. When data distribution is assumed to follow the normal distribution, the *identity-link* function is used and the GLM becomes the linear model for normal data. When data are counts such as number of new cases or number of cumulative cases, the appropriate distribution is the Poisson distribution with the *log-link* function option. When the outcome or response variable is binary, such as whether one is infected with the disease or not, then the appropriate distribution to assume is the binomial distribution with *logit-link* function. ^10, 23^ A detailed discussion of exponential family of distributions and link functions can be found in. ^34, Section 3.1.1^ Estimations of parameters and statistical inferences under the GLMs are based on the theory of maximum likelihood estimation. However maximization of the likelihood requires an iterative least squares approach discussed in. ^34, Section 1.8.8 (p. 54)^ Also see ^34, Section 3.1.2^ for detailed theory on fitting of the Generalized Linear Models.

A GAM is a GLM ith a linear predictor involving a sum of smooth functions of covariates. ^16, 17^ The general form of the GAM is:

$$\begin{array}{l} g(\mu_{i})=H_{i}\theta +f_{1}(x_{1i})+f_{2}(x_{2i})+\ldots +f_{n}(x_{ni}), \end{array}$$ (6)

where *μ _i_* = *E*[ *Y _i_*] and the response variable $Y_{i}∼\exp f(\mu_{i},\phi )$ where $\exp f(\mu_{i},\phi )$ denotes an exponential family distribution with mean *μ _i_* and scale parameter *ϕ.* The variable **H**_*i*_ represents a design matrix of covariates for any strict parametric model components, ***θ*** is a vector of parameter estimates describing the effects of the covariates on the response, *f* are the smooth functions of the covariates *x _k_.* This model introduces flexibility in the specification of the response variable on the covariates. ^34^ However, complications are avoided when the model is specified in terms of “smooth functions” rather than detailed parametric relationships. ^34^ Simon N. ^34^ showed how GAMs can be represented using basis expansions for smooth functions, where each smooth function has an associated penalty controlling function smoothness. Estimation of parameters can be achieved by using penalized regression approaches. The appropriate degree of smoothness for f _j_ can be estimated from data using cross validation or marginal likelihood maximization. ^34^ For univariate smoothing, the representation and estimation of component functions of a model are best introduced taking into account a model consisting of a function of one covariate defined as:

$$\begin{array}{l} y_{i}=f(x_{i})+\epsilon_{i}, \end{array}$$ (7)

where *y _i_* is the response variable, *x _i_* is the covariate, *f* is the smooth function and *ϵ _i_* is random variable defined as *ϵ _i_* ∼ *N*(0, *σ* ^2^). Given equation ( 7), it is possible to represent a function with basis expansions. To estimate *f*, using the approaches applied to linear models, ^34, Chp. 1 and 3^ it is required that *f* be represented such that the function ( 7) becomes a linear model. This can be achieved by selecting a basis that spans the space of functions of *f* or a close approximation to it. The chosen basis functions will be considered as completely known. That is, if *B _j_* ( *x*) is the *j ^th^* basis function, then *f* is assumed to have a representation defined by:

$$\begin{array}{l} f(x)=\overset{k}{\underset{j=1}{\sum }}B_{j}(x)\beta_{j}, \end{array}$$ (8)

where *β _j_* is a vector of unknown parameters. Substituting ( 8) into ( 7) yields:

$$\begin{array}{l} y_{i}=\overset{k}{\underset{j=1}{\sum }}B_{j}(x)\beta_{j}+\epsilon_{i}, \end{array}$$ (9)

which is a linear model.

Suppose that *f* is in the space of fourth order polynomials, then it follows that a basis for this space is

$$B_{1}(x)=1,B_{2}(x)=x,B_{3}(x)=x^{2},B_{4}(x)=x^{3},B_{5}(x)=x^{4}$$

and the equation ( 8) becomes:

$$\begin{array}{l} f(x)=\beta_{1}+x\beta_{2}+x^{2}\beta_{3}+x^{3}\beta_{4}+x^{4}\beta_{5}. \end{array}$$ (10)

and the equation ( 7) becomes the following model:

$$\begin{array}{l} f(x)=\beta_{1}+x\beta_{2}+x^{2}\beta_{3}+x^{3}\beta_{4}+x^{4}\beta_{5}+\epsilon_{i}. \end{array}$$ (11)

In the case of additive models, suppose that there are two covariates, *x* and *v*, describing the changes in a response variable, *y*, then an additive model is defined as:

$$\begin{array}{l} y_{i}=\beta_{0}+f_{1}(x_{i})+f_{2}(v_{i})+\epsilon_{i}, \end{array}$$ (12)

where *β _0_* is the intercept, *f _j_* are the smooth functions, and *ϵ _i_* are independent and identically normally distributed random variable with mean zero and variance *σ ^2^.* A notable issue is that the model now contains more than one function which leads to identifiability issue. It requires identifiability constraints to be imposed on the model before fitting. If the identifiability problem is addressed, then the additive model can be represented using penalized regression splines. ^34, P. 175, Section 4.3.1^ The degree of smoothing is selected by cross validation or (RE)ML as done under the univariate model. Here the basis functions for *f*_1_ are defined by using a sequence of *k*_1_ knots with $x_{j}^{*}$ equally spaced over the domain of *x* and unknown *γ*_j_ coefficients. Also, the basis functions for *f*_2_ are defined by using a sequence of *k*_2_ knots with $v_{j}^{*}$ equally spaced over the range of *v* and unknown *δ _j_* coefficients. It follows that

$$f_{1}={\left[f_{1}(x_{1})+f_{2}(x_{2})+\ldots +f_{n}(x_{n})\right]}^{\prime }$$

and hence

$$f_{1}=X_{1}\gamma ,$$

where the basis *b _j_* ( *x _i_*) is the *i,j* elements of X _1_. On the other hand,

$$f_{2}={\left[f_{1}(v_{1})+f_{2}(v_{2})+\ldots +f_{n}(v_{n})\right]}^{\prime }$$

and hence

$$f_{2}=X_{2}\delta ,$$

where the basis *B _j_* ( *x _i_*) is the *i,j* elements of X _2_. For the identifiability problem, the best constraints according to Simon N. ^34^ are the sum-to-zero constraints:

$$\begin{array}{l} \overset{n}{\underset{i=1}{\sum }}f_{1}(x_{i})=0\;\text{ this is equavalent to}\;\;1^{\prime }f_{1}=0, \end{array}$$ (13)

where 1 ^′^ is a 1 × *n* vector of 1s. This constraint does not change the shape of the smooth function *f*_1_ but shifts *f*_1_ vertically so that the mean value of *f*_1_ is zero. For details on how this constraint can be applied and how additive models can be fitted using penalized least squares, see Simon N. ^34, P. 176-177^

The GAMs are extensions of additive models. Under the GAM framework, the linear predictors predict the known smooth monotonic function of the expected value of the response variable, where the response may follow any exponential family distribution. The linear predictor may simply have a known mean variance relationship which allows for the use of a quasi-likelihood methods. The GAM has the form described in equation ( 6), i.e.:

$$\begin{array}{l} g(\mu_{i})=H_{i}\theta +f_{1}(x_{1i})+f_{2}(x_{2i})+\ldots +f_{n}(x_{ni}). \end{array}$$ (14)

Detailed information on GAM theory can be found in. ^34, Chp. 6^ The GAM is fitted using penalized likelihood maximization, which practically can be achieved by using penalized iterative least squares (PIRLS) described in. ^34, P. 180^

## 3. Results

In this section, linear, polynomial, spline, and GAMs are applied to the COVID-19 data. Under each model framework, the most accurate model is selected and subsequently used for forecasting of the cumulative cases of Covid-19.

### 3.1. Data: COVID-19 Ghana cases

The data used in this study was obtained from the Ghana Health Service and the global cases from the Center for Systems Science and Engineering at Johns Hopkins University. ^31^ The data shows that, as of February 24, 2021, the number of COVID-19 cases registered is about 80,759 with 582 deaths and 73,365 recoveries. ^31^ The left panel of Figure 1 shows the monthly new cases of COVID-19 and the right panel of Figure 1 shows the trend of the number of cumulative cases from March 14, 2020 to February 28 2021. In general, the cumulative number of cases increased over the study period. The new cases registered peaked in July 2020 and then decreased until October 2020. The new cases continued to increase from November 2020 to February 2021 with a sharp increase in January 2021 and a slight decrease in December 2020. This continuous increase in the number of new cases is captured by the curve of cumulative cases.

**Figure 2.**
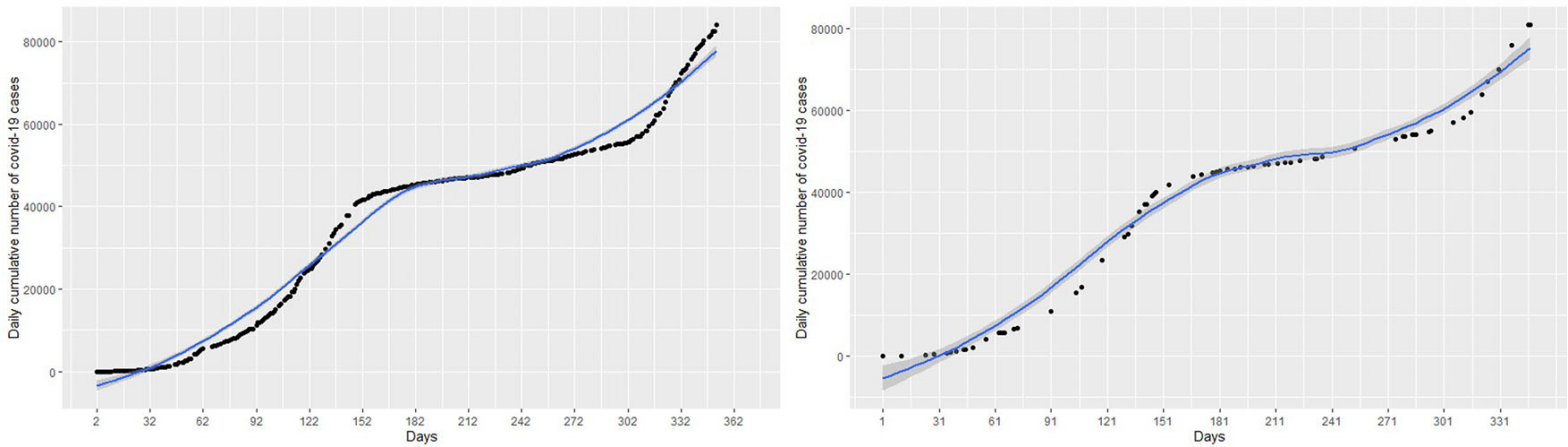
Plots of the training dataset (left panel) and test dataset (right panel) for cumulative coronavirus disease 2019 (COVID-19) cases.

The focus of this study is to determine an appropriate model that can be used to explain the dynamics or trend of cumulative cases and then predict/forecast cumulative cases of the virus for better management decisions. This requires the researcher to find a model that can fit the blue line the data points in the black curve. Statistical models in this work will be implemented for the number of cumulative COVID-19 cases. About 80% of the data was used as training data and the remaining 20% as test data to validate the models. The left panel of the Figure 2 represents the number of cumulative COVID-19 cases for the training dataset and test dataset are presented in the right panel of the Figure 2.

### 3.2. Polynomial modeling of COVID-19 data

Firstly, a naive linear regression model to the cumulative COVID-19 cases in the left panel of Figure 1. The left panel of Figure 1 shows the curve of the linear regression model compared with the real data. This model provides the worst fit with highest RMSE = 6023.14, MAE = 5292.04, MAPE = 28.80654 and R ^2^ = 0.93. The R ^2^ indicates that 93% of the dynamics in the COVID-19 cases have been explained by time because of the general increase in the number of cases. However, the linear model does not capture non-linearity in the data leading to a very high RMSE. This is evident from the left panel of Figure 1, where the predictions of the fitted model (in the blue line) do not follow the observed trend of the COVID-19 cases. Best fit should approximately follow the observed trend shown by the black curve.

**Figure 3.**
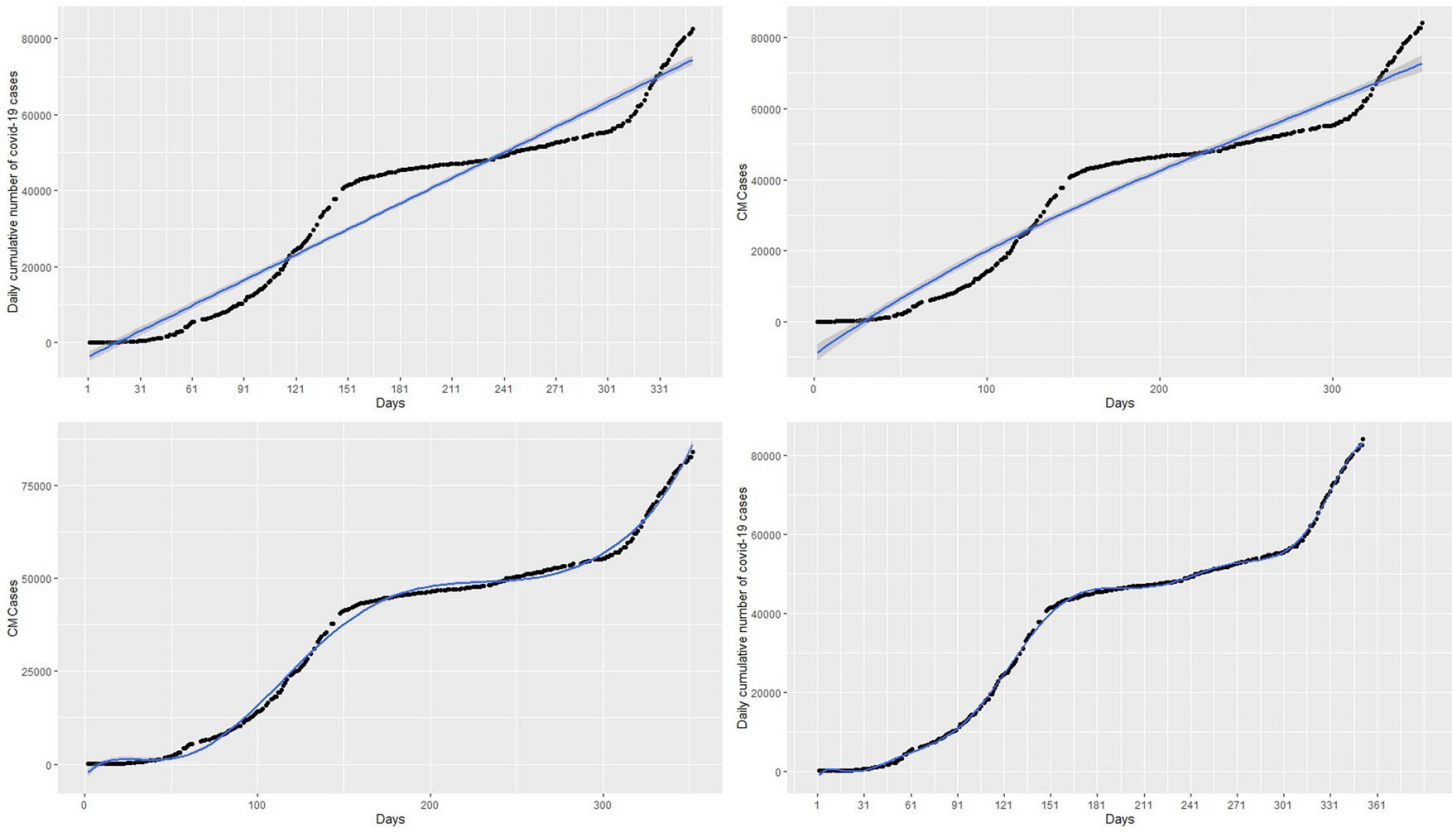
Fits of the linear regression model (top-left panel), polynomial of degree 3 (top-right panel), polynomial of degree 7 (bottom-left panel), and polynomial of degree 11 (bottom-right panel) to the cumulative cases.

Next, a polynomial model with appropriate degree *p* is fitted on the cumulative COVID-19 training dataset in Figure 2 (left panel) and then applied on the test datasets, shown on the right panel of the Figure 2, to validate the model. Various polynomials defined by different degrees *p* were fitted and the polynomial model with degree p = 11 proves to produce the highest R ^2^ and lowest RMSE. The polynomial degrees beyond or below 11 are not significant. That is, polynomials with degree p < 11 produce the highest RMSE and lowest R ^2^ relative to polynomial with degree p = 11. On the other hand, polynomials with degree p > 12 lead to prediction with a rank-deficient fits. The curves of polynomial with degrees 3,7, and 11 are respectively shown in the top-right, bottom-left, and bottom-right panels of the Figure 3. The polynomial with degree 3 has the highest RMSE = 5297.00, MAE = 4914.50, and MAPE = 73.6702 giving the worst fit similar to that of the linear regression model. On the other hand, polynomials with degrees of 7 and 11 appear to provide accurate fits for the cumulative cases but polynomials with degree 7 have a very high RMSE = 1547.25, MAE = 1236.03 and MAPE = 19.67334 relative to RMSE = 591.2077, MAE = 484.9354, MAPE = 7.189698 of the polynomial with degree 11. The polynomial with degree 11 has the highest R ^2^ = 0.999 followed by the degree 7 polynomial (R ^2^ = 0.996) and degree 3 polynomial (R ^2^ = 0.947). The best fitting models from these models are the polynomial with degree 11 since it has the lowest *RMSE* and the highest R ^2^ value (see Table 1). In addition, this model also has the lowest AIC of 4383.862, whereas the polynomials with degree 3 and 7 have AICs of 5687.345 and 4955.772 respectively. Although the polynomial with degree 11 appears to capture the non-linearity in the data, it gives a very poor prediction. This is exhibited in the forecasts in Table 2 and the top-right panel of Figure 6. Although forecasts from the linear model suggest increasing cases (see the top-left panel of the Figure 6), the forecasts from day 1 to day 14 of March 2021 compared with the real data indicate that the linear models are inaccurate for the COVID-19 Ghana data (see Table 1).

**Table 1.** RMSE, R ^2^ and AIC for the Polynomial, Spline and GAM models.

Model	RMSE	R ^2^	AIC	MAE	MAPE
Polynomial	693.7195	0.9995	4383.862	484.9354	7.189698
Spline	296.2845	0.9998	3959.921	356.5673	1.234563
GAM	694.8442	0.9990	4465.724	584.8418	2.065671

**Table 2.** Forecasts of cumulative COVID-19 cases from March 1 to March 31, 2021.

Day-March	Linear model	Polynomial model	Spline model	GAM
1	75335.29	83213.59	83860.91	86402.72
2	75560.03	83309.33	84274.59	87183.94
3	75784.76	83337.86	84676.05	87965.16
4	76009.50	83296.45	85065.62	88746.38
5	76234.24	83182.63	85443.63	89527.60
6	76458.98	82994.21	85810.43	90308.82
7	76683.72	82729.32	86166.33	91090.05
8	76908.45	82386.47	86511.68	91871.27
9	77133.19	81964.62	86846.82	92652.49
10	77357.93	81463.16	87172.06	93433.71
11	77582.67	80882.05	87487.75	94214.93
12	77807.41	80221.82	87794.22	94996.15
13	78032.14	79483.67	88091.81	95777.37
14	78256.88	78669.51	88380.84	96558.59
15	78481.62	77782.06	88661.65	97339.81
16	78706.36	76824.90	88934.57	98121.04
17	78931.10	75802.56	89199.95	98902.26
18	79155.83	74720.60	89458.10	99683.48
19	79380.57	73585.71	89709.37	100464.70
20	79605.31	72405.79	89954.09	101245.92
21	79830.05	71190.05	90192.59	102027.14
22	80054.79	69949.15	90425.20	102808.36
23	80279.53	68695.23	90652.26	103589.58
24	80504.26	67442.13	90874.11	104370.81
25	80729.00	66205.41	91091.07	105152.03
26	80953.74	65002.55	91303.48	105933.25
27	81178.48	63853.05	91511.67	106714.47
28	81403.22	62778.59	91715.98	107495.69
29	81627.95	61803.14	91916.74	108276.91
30	81852.69	60953.17	92114.28	109058.13
31	82077.43	60257.75	92308.94	109839.35

### 3.3. Spline modeling of COVID-19 Data

Again we fit a spline model with appropriate knots and degree of polynomial to the cumulative COVID-19 training dataset in in the left panel of Figure 2 and then use the test datasets in the right panel of Figure 2 to evaluate the fitted spline model. This checks the ability of the fitted spline model to capture and explain the non-linearity in the COVID-19 cases. This means that we have specify two parameters include the degree of polynomial and the location of the knots. ^7^ Following ^7^ example, we have to chose values between 0.20 and 0.95 quantiles as the knots. Choosing and placing three knots at the lower, median, and upper quartiles produced a very bad fit of the data. In fact, we need to identify at least 14 knots between 0.20 and 0.95 quantiles for placement rather knots at the lower, median, and upper quartiles in Bruce and Bruce’s example. ^7^

**Figure 4.**
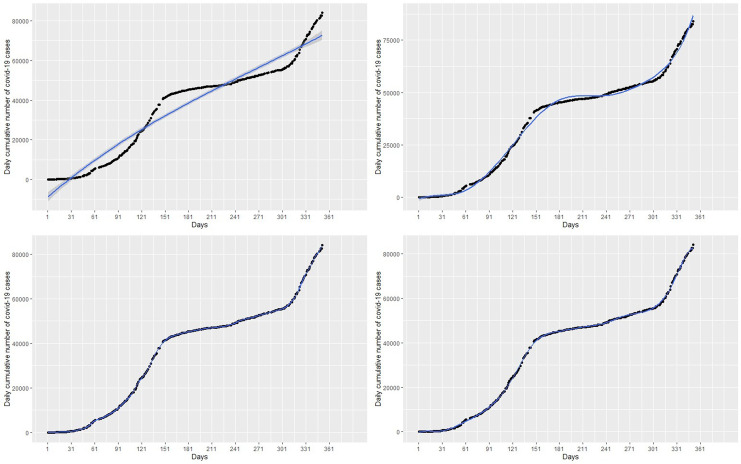
Fit of the spline regression models with 3 knots (top-left panel), 8 knots (top-right panel), 14 knots (bottom-left panel), and 50 knots (bottom-right panel) to the cumulative cases.

The spline model with 3 knots or degrees of freedom (df) which poorly fit the data are shown in the top-left panel of Figure 4. We observed that knots of less than 14 do not provide a best fit for the data with relatively high RMSE. For instance, a spline fit with 3 knots in the top-right panel of Figure 4 and 8 knots in the top-left panel of Figure 4B poorly fit the data. However, knots greater than or equal to 14 provide the best fit of the data with relatively low RMSE and AIC, MAE, and MAPE as shown in Table 1. For example, the bottom-left panel of Figure 4 and the bottom-right panel of Figure 4, with knots 14 and 50 respectively, appear to provide the best fit for the data. The spline model provides predictions almost exactly the same as the original data. This is exhibited in the forecast values in Table 2, where forecasts and observed cases for 1 to 8 March are almost the same and showed a general increase in the covid-19 cases from 1 March to 31 March 2021 in the bottom-left panel of Figure 6. The green dots in Figure 7 show an increasing trend of the observed COVID-19 cumulative cases in March which support the forecasts produced by this model.

### 3.4. GAM for COVID-19

The *gam* function from *mgcv* package in R software was used to implement the GAM. The *gam* model formulation allows for the inclusion of smooth terms such as splines *s()* and tensor products *te().* In the *gam* function, there are a number of options available for controlling automatic smoothing parameter estimation. ^34^

**Figure 5.**
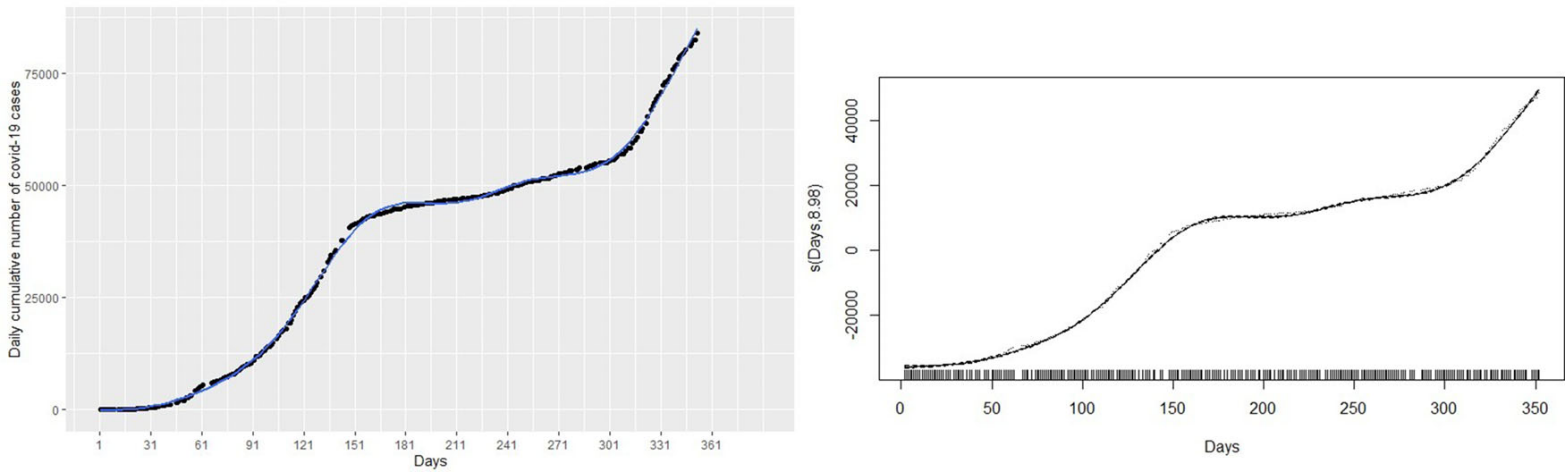
Fit of the generalized additive model (left panel) with the effect of time (days) estimated as a smooth curve with 8.98 degrees of freedom and a plot of days versus partial residuals (right panel).

The left panel of Figure 5 presents the plot of the fitted GAM to the COVID-19 cumulative cases. It can be observed that the GAM is able to capture the non-linearity exhibited by the COVID-19 cases. The effect of time (Days) is estimated as a smooth curve with 8.98 degrees of freedom and the p −value associated spline term s(Days) is less than 0.05 which gives an indication that time in days has significant effect on COVID-19 cases. The effective degrees of freedom (edf) is approximately 9 indicating that polynomial of degree 9 can be used for predicting. The total degrees of freedom is 9.98. The right panel of Figure 5 shows the plot of partial residuals:

$${\hat{\epsilon }}_{i}^{\text{partial}}=f({\text{Days}}_{i})+{\hat{\epsilon }}_{i}^{\text{p}}$$

versus time (days). The right panel of Figure 5 shows that the estimated effect of days with a corresponding 95% confidence intervals is strictly Bayesian credible intervals ^34, P. 293^ shown as dashed lines. The points where the confidence limits and the fitted curve pass through zero on the vertical axis are due to the identifiability constraints imposed to smoothen the time (Days) term. From the right panel of Figure 5, it can be observed that the partial residuals are uniformly scatted round the fitted curve. This gives an indication that the model describes the data well.

**Figure 6.**
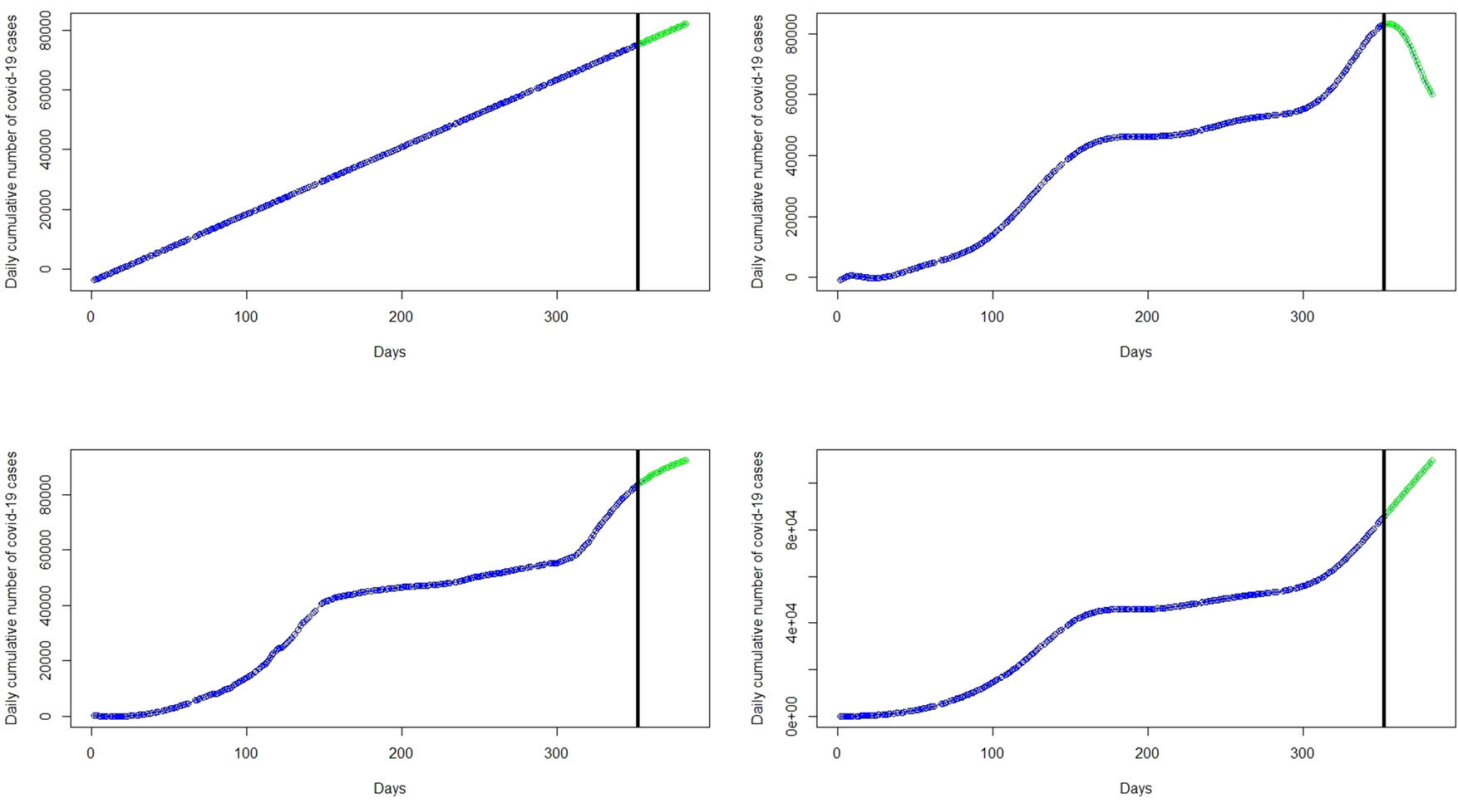
Plots of the observed data values (blue marker dots) and forecasts (green marker dots) of cumulative coronavirus disease 2019 (COVID-19) cases using linear regression (top-left panel), polynomial regression (top-right panel), spline regression (bottom-left panel), and generalized additive model (bottom-right panel).

**Figure 7.**
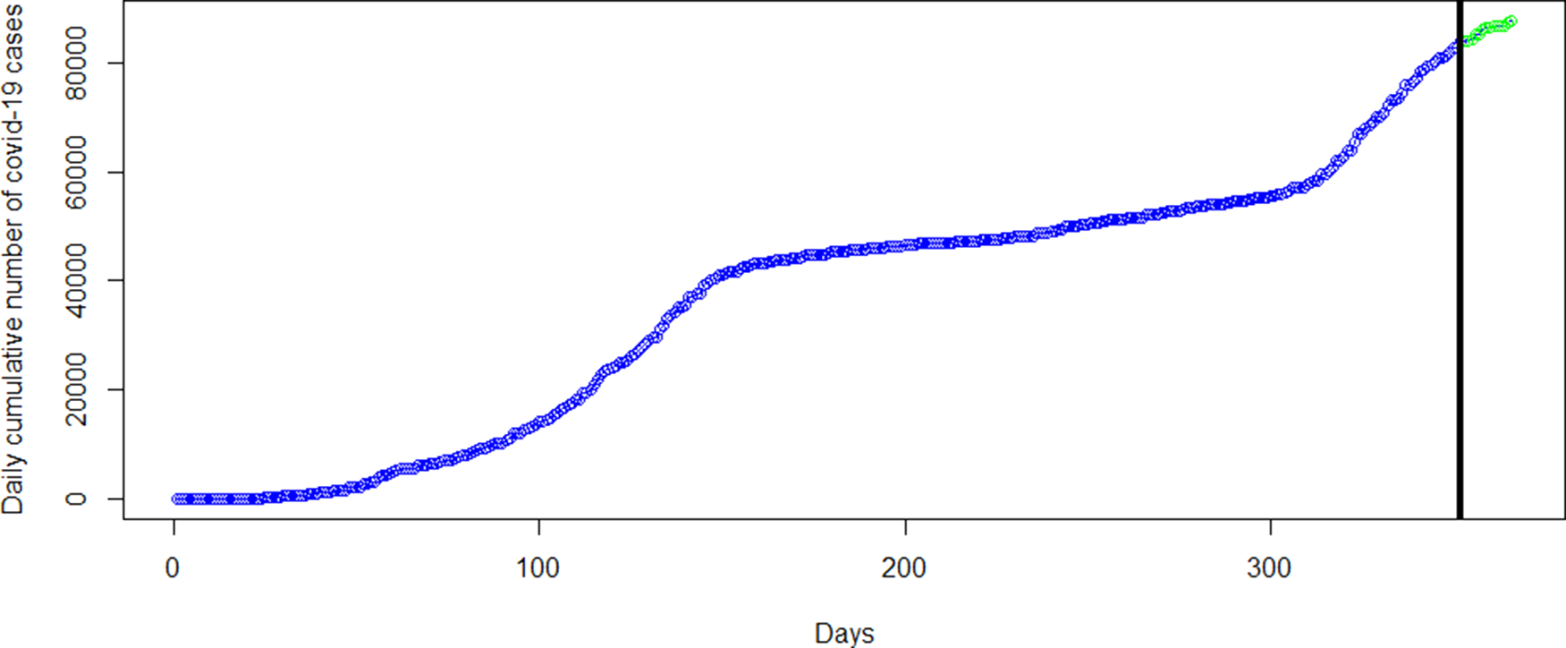
Observed COVID-19 data used in analysis from March 14, 2020 to February 28, 2021 (blue marker dots) with forecast data from day 1 to day 31 of March, 2021 (green marker dots).

The GAM model provides predictions similar to the original data. This observation is shown in the forecast values in Table 2, where forecasts and real data values from day 1 to day 8 of March 2021 are almost the same and show an increase in the COVID-19 cases (see the bottom-right panel of the Figure 6). The green dots in Figure 7 shows an increasing trend of cumulative COVID-19 cases in March which supports the forecasts produced by this model.

### 3.5. Forecasting of cumulative COVID-19 Cases

In this section, the most accurate polynomial, spline and GAM regression models are applied to forecast the number of cumulative COVID-19 cases for one month (from 1 March 2021 to 31 March 2021). Figure 6 presents plots of the forecasted cumulative COVID-19 cases from 1 March (353 days) to 31 March (383 days) 2021.

## 4. Discussion and conclusion

In this work, the dynamics of cumulative COVID-19 cases in Ghana have been modelled. The trend of COVID-19 cases is non-linear, thus, the goal is to determine an appropriate predictive model for forecasting COVID-19 cases in Ghana. The non-linearity implies that simple linear regressions are not accurate, therefore, cannot be used for predicting and forecasting the COVID-19 cases. However, polynomials, splines, and GAMs have the ability to capture non-linearity. Thus, such models have been developed for forecasting cumulative COVID-19 cases in Ghana. About 80% of the real data was used for training the models and the remaining 20% used for model validation. Data analyses was carried out with the aid of the *R* software. ^29^

Further, many polynomials, splines and GAMs were applied to the COVID-19 data and RMSE), AIC, and R-square (R ^2^), MAE, and MAPE were used to determine the most accurate models (models with the lowest RMSE, lowest AIC, and the highest R ^2^ are the most accurate) in each category. Among the polynomial models, those with degree 11 (see the bottom-right panel of Figure 3) provided the best fit. Among the spline models, those with knots greater than or equal to 14 (see bottom-left panel of Figure 4 and bottom-right pane of Figure 4) provided accurate fits for the data. The GAMs with time estimated as a smooth curve with 8.98 degrees of freedom (see the right panel of Figure 5) were very accurate for the cumulative COVID-19 cases.

Moreover, the most accurate models were then used to forecast cases for the entire month of March, 2021. The forecasts from each category of models are shown in Figure 6 with the green marker dots. The linear regression model obviously does not fit the data well and hence, the forecasts for March 2021 are far from what has been observed (see Table 2 and Figure 7). Although the polynomial model fits the data well (see the bottom-right panel of Figure 3), it provides inaccurate forecasts for March 2021 (see the top-right panel of Figure 6, Table 2 and Figure 7). The spline model and the GAM provide accurate forecast values for March 2021. This finding is in line with the literature on Splines and GAM models in relation to their ability to provide best fit to complex non-linear data points, especially GAM. ^45– 48^ In the GAM framework, one is able avoid overfitting by controlling the smoothness of the predictor functions. The GAM framework uses automatic smoothness selection approaches in order determine the complexity of the fitted trend and also provides a framework for potentially complex and non-linear trends. ^48^ Overfitting is avoided by accounting for model uncertainty and the identification of time points with significant temporal change. ^48^

The aim of this research is to provide guide to decision-making authorities so that necessary measures can be taken timely and effectively to avoid or slow the spread of COVID-19. Our study results revealed that cumulative COVID-19 cases in Ghana are expected to continue to increase if appropriate preventive measures are not enforced. We therefore recommend strict observance of all COVID-19 protocol measures proposed by the health authorities. Also, government and stakeholders should be prepared to allocate more resources for the effective management of the virus. The forecast provided in this paper is vital for proper management of the covid-19 virus so as to enhance decision-making and reduce the spread of the virus in Ghana.

Ghana is a developing country with inadequate health facilities and personnel making it difficult in fighting the spread of the virus. Hence, though decisions should be adopted by government officials and public health worker in other to reduce the spread of the COVID-19. On the other hand, citizens must strictly observe all protocol measures to control the spread of the virus.

Vaccination against the virus is ongoing in Ghana, thus, future research would consider evaluating the impact of the vaccine.

## Data availability

The datasets analyzed in this study can be found at the [Center for Systems Science and Engineering at Johns Hopkins University] [ https://www.statista.com/statistics/1110892/coronavirus-cumulative-cases-in-ghana/].
