# Supplementary material for: A predictive model for daily cumulative COVID-19 cases in Ghana
Source: F1000Res. 2022 Mar 4;10:343. Originally published 2021 May 5. [Version 2] doi: 10.12688/f1000research.52403.2 (PMC9005990; doi:10.12688/f1000research.52403.2)
Supplement: Peer review history

# Peer review: Reviewer response for version 2

**DOI:** 10.5256/f1000research.121904.r126263

**Published:** 2022-04-12

**Authors:** Muhammad Aamir

**Affiliations:** 1 Department of Statistics, Abdul Wali Khan University, Mardan, Mardan, Pakistan

All of my comments of the previous round have been incorporated. Now I am suggesting Approving the paper in current form.

Is the work clearly and accurately presented and does it cite the current literature?

Yes

If applicable, is the statistical analysis and its interpretation appropriate?

Yes

Are all the source data underlying the results available to ensure full reproducibility?

Yes

Is the study design appropriate and is the work technically sound?

Partly

Are the conclusions drawn adequately supported by the results?

Yes

Are sufficient details of methods and analysis provided to allow replication by others?

Yes

Reviewer Expertise:

Time series analysis, Machine learning, and statistical modeling

I confirm that I have read this submission and believe that I have an appropriate level of expertise to confirm that it is of an acceptable scientific standard.

# Peer review: Reviewer response for version 2

**DOI:** 10.5256/f1000research.121904.r126264

**Published:** 2022-03-04

**Authors:** Nicola Bartolomeo

**Affiliations:** 1 Interdisciplinary Department of Medicine - Section of Hygiene and Medical Statistics, University of Bari Aldo Moro, Bari, Italy

The authors accepted the suggestions and the manuscript can be accepted for indexing

Is the work clearly and accurately presented and does it cite the current literature?

Partly

If applicable, is the statistical analysis and its interpretation appropriate?

Yes

Are all the source data underlying the results available to ensure full reproducibility?

Yes

Is the study design appropriate and is the work technically sound?

Yes

Are the conclusions drawn adequately supported by the results?

Yes

Are sufficient details of methods and analysis provided to allow replication by others?

Yes

Reviewer Expertise:

Biostatistics; epidemiology; public health

I confirm that I have read this submission and believe that I have an appropriate level of expertise to confirm that it is of an acceptable scientific standard.

# Peer review: Reviewer response for version 1

**DOI:** 10.5256/f1000research.55677.r99083

**Published:** 2021-11-08

**Authors:** Nicola Bartolomeo

**Affiliations:** 1 Interdisciplinary Department of Medicine - Section of Hygiene and Medical Statistics, University of Bari Aldo Moro, Bari, Italy

The authors aim to explain the pattern of growth in the number of cumulative cases of COVID-19 and also predict the number of cumulative cases in Ghana. The paper is interesting as linear, polynomial and generalized (GLM) models are used after reviewing the main forecasting models used since the Covid-19 pandemic began. The paper is quite complete in its introduction, methods and results, while the discussion appears repetitive and incomplete.

**Major revisions**

1. In the methods section a sensitivity analysis should be proposed, testing the parameters estimated with the three models in pandemic periods subsequent to the one analyzed.
2. The discussion section should be rewritten and expanded. Avoid referring to results and figures already presented in the previous section. Rather, the discussion must arise from the comparison between the results obtained with one's own work with respect to the existing literature, in terms of the efficiency of the parameters, the quality of the estimates and the precision of the results on the forecasts.

**Minor revisions**

1. In the methods section, and consequently in the results section, further possible measures of goodness of fitting should be included, useful for comparing the proposed models, such as mean absolute error (MAE) and mean absolute percentage error (MAPE).
2. In a study recently published in the journal "Infectious disease modeling" Bartolomeo *et al.* (2021) ^1^ have shown that an exponential decay model applied to the weighted and averaged growth rates appears to be better than growth models such as Gompertz's for modeling the number of cases of the COVID-19. Discuss about this.

Is the work clearly and accurately presented and does it cite the current literature?

Partly

If applicable, is the statistical analysis and its interpretation appropriate?

Yes

Are all the source data underlying the results available to ensure full reproducibility?

Yes

Is the study design appropriate and is the work technically sound?

Yes

Are the conclusions drawn adequately supported by the results?

Yes

Are sufficient details of methods and analysis provided to allow replication by others?

Yes

Reviewer Expertise:

Biostatistics; epidemiology; public health

I confirm that I have read this submission and believe that I have an appropriate level of expertise to confirm that it is of an acceptable scientific standard, however I have significant reservations, as outlined above.

## References

[1] : Short-term forecast in the early stage of the COVID-19 outbreak in Italy. Application of a weighted and cumulative average daily growth rate to an exponential decay model. *Infect Dis Model* .2021;6: 10.1016/j.idm.2020.12.007 212-221 10.1016/j.idm.2020.12.007 33398249PMC7773318

# Response

**Published:** 2022-02-28

**Authors:** Abdul-Karim Iddrisu

**Affiliations:** University of Energy and Natural Resources, Ghana

Reviewers' report

 Manuscript ID: 52403

 Title: A predictive model for daily cumulative COVID-19 cases in Ghana

 We would like to thank the reviewers for the many useful and insightful comments. We have now revised the manuscript substantially. We trust that the revised manuscript now meets your required standards for indexing.

 Please find below our response (highlighted or tracked in the manuscript) to the reviewers’ comments in the revised manuscript.

***Comment 1:*** *The discussion section should be rewritten and expanded. Avoid referring to results and figures already presented in the previous section. Rather, the discussion must arise from the comparison between the results obtained with one's own work with respect to the existing literature, in terms of the efficiency of the parameters, the quality of the estimates and the precision of the results on the forecasts.*

**Response 1** *:  *We thank the reviewer for these useful comments. In response,

*“*This finding is in line with the literature on Splines and GAM models in relation to their ability to provide best fit to complex non-linear data points, especially GAM ^10–13^. In the GAM framework, one is able avoid overfitting by controlling the smoothness of the predictor functions.  The GAM framework uses automatic smoothness selection approaches in order determine the complexity of the fitted trend and also provides a framework for potentially complex and non-linear trends ^13^. Overfitting is avoided by accounting for model uncertainty and the identification of time points with significant temporal change ^13^.”

***Comment 2:*** *In the methods section, and consequently in the results section, further possible measures of goodness of fitting should be included, useful for comparing the proposed models, such as mean absolute error (MAE) and mean absolute percentage error (MAPE).*

**Response 2:** Thank you for these comments. We have now revised the manuscript to reflect MAE and MAPE in the methods and results Sections 3 and 4.

***Comment 3:*** *In a study recently published in the journal "Infectious disease modeling" Bartolomeo et al. (2021)* *^1^* *have shown that an exponential decay model applied to the weighted and averaged growth rates appears to be better than growth models such as Gompertz's for modeling the number of cases of the COVID-19. Discuss about this.*

**Response 3: **We thank the reviewer for this important recommendation/suggestion. In response, we have discussed the *Bartolomeo et al. (2021) paper in the introductory Section 1.*

# Peer review: Reviewer response for version 1

**DOI:** 10.5256/f1000research.55677.r88360

**Published:** 2021-08-02

**Authors:** Muhammad Aamir

**Affiliations:** 1 Department of Statistics, Abdul Wali Khan University, Mardan, Mardan, Pakistan

The study “A predictive model for daily cumulative COVID-19 cases in Ghana” is interesting. In this work, the dynamics of cumulative COVID-19 cases in Ghana have been modelled. The trend of COVID-19 cases is non-linear, thus, the goal is to determine an appropriate predictive model for forecasting COVID-19 cases in Ghana. The study was mostly focused on the comparison of linear and non-linear models. The paper is well set, and the contents are clearly described. The authors almost achieved their objectives. However, the following suggestions should be incorporated before resubmitting the paper:

1. A section of “conclusion” must be added, which concludes the whole paper and provides some suggestions for the government to make decisions. For writing the conclusion, take help from this paper of mine ^1^.
2. Please provide some details that explain why cumulative cases are considered in this study. Most of the studies conducted on the daily cases but not cumulative - is there any specific reason? The authors must indicate.
3. At some places, the use of Figures is mixed which needs rectification. Especially Figure 6 and Figure 7.
4. Check the caption of Figure 7. It is March 14 or March 31?

Is the work clearly and accurately presented and does it cite the current literature?

Yes

If applicable, is the statistical analysis and its interpretation appropriate?

Yes

Are all the source data underlying the results available to ensure full reproducibility?

Yes

Is the study design appropriate and is the work technically sound?

Partly

Are the conclusions drawn adequately supported by the results?

Yes

Are sufficient details of methods and analysis provided to allow replication by others?

Yes

Reviewer Expertise:

Time series analysis, Machine learning, and statistical modeling

I confirm that I have read this submission and believe that I have an appropriate level of expertise to confirm that it is of an acceptable scientific standard, however I have significant reservations, as outlined above.

## References

[1] : Analysis and Forecasting COVID-19 Outbreak in Pakistan Using Decomposition and Ensemble Model. *Computers, Materials & Continua* .2021;68(1) : 10.32604/cmc.2021.012540 841-856 10.32604/cmc.2021.012540

# Response

**Published:** 2022-02-28

**Authors:** Abdul-Karim Iddrisu

**Affiliations:** University of Energy and Natural Resources, Ghana

Reviewers' report

 Manuscript ID: 52403

 Title: A predictive model for daily cumulative COVID-19 cases in Ghana

 We would like to thank the reviewers for the many useful and insightful comments. We have now revised the manuscript substantially. We trust that the revised manuscript now meets your required standards for indexing.

 Please find below our response (highlighted or tracked in the manuscript) to the reviewers’ comments in the revised manuscript.

***Comment 1*** *: A section of “conclusion” must be added, which concludes the whole paper and provides some suggestions for the government to make decisions. For writing the conclusion, take help from this paper of mine*

**Response 1: **We thank the reviewer for these comments. In response, we have added conclusion as,

 “This finding is in line with the literature on Splines and GAM models in relation to their ability to provide best fit to complex non-linear data points, especially GAM ^10–13^. In the GAM framework, one is able avoid overfitting by controlling the smoothness of the predictor functions. The GAM framework uses automatic smoothness selection approaches in order determine the complexity of the fitted trend and also provides a framework for potentially complex and non-linear trends ^13^. Overfitting is avoided by accounting for model uncertainty and the identification of time points with significant temporal change ^13^.

 The aim of this research is to provide guide to decision-making authorities so that necessary measures can be taken timely and effectively to avoid or slow the spread of COVID-19. Our study results revealed that cumulative COVID-19 cases in Ghana are expected to continue to increase if appropriate preventive measures are not enforced. We therefore recommend strict observance of all COVID-19 protocol measures proposed by the health authorities. Also, government and stakeholders should be prepared to allocate more resources for the effective management of the virus. The forecast provided in this paper is vital for proper management of the covid-19 virus so as to enhance decision-making and reduce the spread of the virus in Ghana.

 Ghana is a developing country with inadequate health facilities and personnel making it difficult in fighting the spread of the virus. Hence, though decisions should be adopted by government officials and public health worker in other to reduce the spread of the COVID-19. On the other hand, citizens must strictly observe all protocol measures to control the spread of the virus.”

***Comment 2*** *: Please provide some details that explain why cumulative cases are considered in this study. Most of the studies conducted on the daily cases but not cumulative - is there any specific reason? The authors must indicate.*

**Response 2: **Thank you for the comments. In response, we note that,

**“**Various authors have modeled, predicted and forecast cumulative cases of COVID-19 to study the dynamics of cumulative cases over a period of time ^2–7^. The authors in ^7^ used cumulative covid-19 data and time series models to forecast the epidemiological trends of COVID-19 pandemic for top-16 countries where 70%–80% of global cumulative cases are high. Also, a deep learning ensemble approach has been adapted by the authors in ^6^ to determine the best auto-regressive integrated moving average (ARIMA) model for predicting and forecasting cumulative COVID-19 cases across multi-region countries. Nonlinear growth models such as the Gompertz, Richards, and Weibull were implemented to cumulative covid-19 data in order to study the daily cumulative number of COVID-19 cases in Iraq ^5^. Bartolomeo *et al*. ^8^ applied the exponential decay model (EDM) to estimate and forecast the cumulative number of COVID-19 infections in Italy. These authors compared the EDM and the Gompertz model. The exponential decay model applied to the weighted and averaged growth rates appears to be better than growth models such as Gompertz's for modeling the number of cases of the COVID-19. In this study, linear, polynomial and generalized linear models (GLMs) are employed to explain the growth pattern of the number of cumulative cases of COVID-19 and also, to predict and forecast the number of cumulative cases in Ghana. These models were implemented to the Ghana COVID-19 data and compared for best model selection and results discussed and conclusion drawn.”

***Comment 3:*** *At some places, the use of Figures is mixed which needs rectification. Especially Figure 6 and Figure 7*. *Check the caption of Figure 7. It is March 14 or March 31?*

**Response 3: **The caption of Figure 7 should read,

 "Observed COVID-19 data used in analysis from March 14, 2020 to February 28, 2021 (blue marker dots) with forecast data from day 1 to day 31 of March, 2021 (green marker dots).”
